# Default-Mode Network Connectivity Changes Correlate with Attention Deficits in ALL Long-Term Survivors Treated with Radio- and/or Chemotherapy

**DOI:** 10.3390/biology11040499

**Published:** 2022-03-24

**Authors:** Federica Mazio, Giuseppina Aloj, Grazia Maria Giovanna Pastorino, Teresa Perillo, Carmela Russo, Maria Pia Riccio, Eugenio Maria Covelli, Rosanna Parasole, Enrico Tedeschi, Lorenzo Ugga, Alessandra D’Amico, Mario Quarantelli

**Affiliations:** 1 Pediatric Neuroradiology, Department of Neuroscience, Santobono-Pausilipon Children’s Hospital, 80129 Naples, Italy; federicamazio1@gmail.com (F.M.); russocarmela84@gmail.com (C.R.); e.covelli@santobonopausilipon.it (E.M.C.); 2 Department of Pediatric Hemato-Oncology, A.O.R.N. Santobono-Pausilipon, 80123 Naples, Italy; giuseppinaaloj@gmail.com (G.A.); rparasol64@gmail.com (R.P.); 3 Child and Adolescent Neuropsychiatry Unit, Department of Medicine, Surgery and Dentistry, University of Salerno, 84084 Salerno, Italy; graziapastorino@gmail.com; 4 Department of Advanced Biomedical Sciences, University of Naples Federico II, 80131 Naples, Italy; tperillo3@gmail.com (T.P.); enrico.tedeschi@unina.it (E.T.); lorenzo.ugga@gmail.com (L.U.); 5 Department of Medical and Translational Sciences, Child Neuropsychiatry, Federico II University, Via Pansini 5, 80131 Naples, Italy; piariccio@gmail.com; 6 Department of Radiology, Tortorella Private Hospital, 84124 Salerno, Italy; damicoalex@tiscali.it; 7 Institute of Biostructure and Bioimaging, National Research Council, 80145 Naples, Italy

**Keywords:** long-term survivors, acute lymphoblastic leukemia, MRI, brain, resting-state fMRI, default-mode network, voxel-based morphometry

## Abstract

**Simple Summary:**

Both chemotherapy and radiotherapy play a role in the neurocognitive impairment of long-term survivors from acute lymphoblastic leukemia, but it is unknown if similar mechanisms are involved. We assessed neurocognitive alterations, brain tissue volumes, and functional connectivity of the main hubs of the default-mode network, in 13 patients treated with chemotherapy and radiotherapy (Group A) and in 13 treated with chemotherapy only (Group B). Correlations with neuropsychological scores, independent of group, were assessed for regions that showed significant differences between the two groups at neuroimaging. Compared to Group B, Group A performed significantly worse at the digit span and digit symbol tests and showed increased functional connectivity between the medial prefrontal cortex and the rolandic operculi, along with the absence of differences in regional brain tissue volumes. Functional connectivity in these regions correlated inversely with speed of processing in both groups, suggesting that similar mechanisms may be involved in the neurocognitive deficits in both groups.

**Abstract:**

Whether chemotherapy (ChT) and radiotherapy (RT) determine neurocognitive impairment in acute lymphoblastic leukemia long-term survivors (ALL LTSs) through similar mechanisms affecting the same brain regions is still unknown. We compared neurocognitive alterations, regional brain tissue volumes (by voxel-based morphometry), and functional connectivity of the main default-mode network hubs (by seed-based analysis of resting state functional MRI data), in 13 ALL LTSs treated with RT and ChT (Group A) and 13 treated with ChT only (Group B). Group A performed significantly worse than Group B at the digit span and digit symbol tests (*p* = 0.023 and 0.013, respectively). Increased connectivity between the medial prefrontal cortex (the main anterior hub of the default-mode network) and the rolandic operculi was present in Group A compared to Group B, along with the absence of significant differences in regional brain tissue volumes. In these regions, the functional connectivity correlated inversely with the speed of processing scores, independent of treatment group. These results suggest that similar mechanisms may be involved in the neurocognitive deficits in ALL LTS patients, regardless of the treatment group. Further studies are needed to clarify whether these changes represent a direct expression of the mechanisms underlying the cognitive deficits or ineffective compensatory phenomena.

## 1. Introduction

Acute lymphoblastic leukemia (ALL) is a hematologic malignancy that represents the most common pediatric cancer, with an estimated age-standardized incidence rate (per 100,000 individuals) of 0.85 worldwide [1]. The central nervous system (CNS) is involved in almost 5–8% of cases [2]. The mainstay of ALL treatment has moved from cranial radiation to high-dose system chemotherapy plus intrathecal long-term chemotherapy [3,4]. Due to the improvement of treatment protocols, the population of ALL long-term survivors (LTS) has progressively increased in the last decades. ALL LTSs have higher risk of morbidity and mortality compared to the general population [5,6]. Brain radiotherapy (RT) has both subacute and chronic negative effects on the central nervous system that can get worse over time [7], affecting attention and memory [8,9], with a prevalence of cognitive impairment up to 32% (depending on the specific function and radiation dose) [9,10]. On the other hand, neurocognitive impairment has also been found in 10–25% of ALL LTSs treated with chemotherapy (ChT) only [11,12], with attention and memory deficits being again the most frequent findings [13,14]. Accordingly, neuropsychological assessment is currently recommended in patients undergoing both treatments [15].

Brain is a very efficient network made up of a large number of regions differently distributed in space, each with a specific task, but functionally connected to each other for a continuous exchange of information [16]. Resting-state functional MRI (RS-fMRI) is a technique capable of assessing the integrity of this functional brain architecture, probing functional connectivity (FC), which can be defined as the synchronous neuronal activity between different brain regions that characterize spontaneous neuronal activity in resting conditions [17].

Thanks to RS-fMRI, specific networks, characterized by distributed synchronous neuronal activity patterns, have been identified. Among these, the default mode network (DMN) is the most widely investigated in the literature, due to its central role as the main task-negative network. DMN indeed interacts closely with all the main task-positive networks [18,19], and alterations of the DMN have been shown in a large host of neurological and psychiatric pathologies [20], as well as in ALL LTS treated with ChT [21,22]. Considering the still limited information available on the brain areas and mechanisms involved in the neurocognitive impairment in ALL LTS, and on the mechanisms underlying the effects of the different therapeutic regimens, our study aims were (1) to evaluate neurocognitive alterations in two groups of ALL LTSs (off-therapy for at least 2 years), treated with ChT and RT or with ChT alone, (2) to compare regional gray- (GM) and white-matter (WM) volumes between the two groups using voxel-based morphometry (VBM), (3) to compare FC changes of the main anterior and posterior DMN hubs in the two groups using RS-fMRI, and (4) to correlate neuroimaging results to neurocognitive performances.

## 2. Materials and Methods

### 2.1. Subjects

In this controlled observational cross-sectional study, we selected ALL LTS patients from a cohort of 334 patients treated in the Pediatric Hemato-Oncology Department of the Santobono-Pausilipon Hospital in Naples, Italy. Inclusion criteria were as follows: diagnosis of ALL; age > 8 years old; treatment in accordance with the 1995–2009 protocols for clinical trials of the Italian Association of Pediatric Hematology and Oncology (Associazione Italiana di Ematologia ed Oncologia Pediatrica—AIEOP) and the AIEOP–Berlin–Frankfurt–Münster consortium (AIEOP-BFM); off therapy for at least 2 years; good compliance with neurocognitive tests; subscription of written informed consent. Exclusion criteria were as follows: contraindications to MRI; suboptimal compliance to neurocognitive tests; relapsed disease; hematopoietic stem-cell transplantation; preexisting medical or psychiatric conditions affecting cognitive assessment. Among the eligible patients, 43 were treated with both RT and systemic and intrathecal ChT. Of these, 25 did not meet the inclusion/exclusion criteria, and five refused to participate, leaving 13 patients in Group A (administered doses were 24 Gy in one patient with CNS extended disease at onset, 18 Gy prophylactic dose in the remaining 12 patients). Among the remaining eligible ALL LTSs, who were treated only with systemic and intrathecal ChT, 13 patients (Group B) were matched pairwise with the patients of Group A. Matching criteria were chosen to minimize pairwise differences in age, sex, ethnicity, scholarity, handedness, use of eyeglasses, age at LLA diagnosis, years from the end of the treatment, and type of therapeutic protocol (apart from the use of RT). Table 1 summarizes the demographic information of the enrolled subjects. Details of ALL subtypes and karyotypes are provided in Supplementary Table S1. Details of intrathecal and i.v. administrations of methothrexate (the unique chemotherapeutic agent included in these protocols which is able to cross the BEE) are provided in Supplementary Table S2. One patient from Group A underwent neurocognitive tests but refused MRI, while one MRI exam from Group B was discarded due to excessive movements (see below). The study was conducted in compliance with the ethical standard and approved by the local Ethics Committee “A. Cardarelli/Santobono-Pausilipon” (Prot. 72/17, 18 June 2020). Written informed consent was obtained from all subjects according to the Declaration of Helsinki.

### 2.2. Clinical and Neurocognitive Evaluation

All patients underwent the following standardized tests:

1. The Wechsler Adult Intelligence Scale Revised (WAIS-R) for patients older than 16 years and the Wechsler Intelligence Scale for Children (WISC-IV) for patients below 16 years of age, for cognitive evaluation [23,24]. We considered only the scores common to both scales: total intelligence quotient (TIQ; mean = 100, SD = 15); digit span, digit symbol/coding, block design, vocabulary, comprehension, similarities (mean = 10, SD = 3 for all). Standard scores < 1 SD are considered at the low limits of the norm.
2. D2-R Test of Attention (d2-R) [25]. It assesses selective and sustained attention and visual scanning speed through three parameters: processing speed, processing accuracy, and error rate. Standard scores ≤ 94 are considered at the low limits of the norm.
3. Wisconsin Card Sorting Test (WCST) [26]. It measures working memory, problem-solving strategies, and frontal lobe damage. We considered total errors, perseverative answers, perseverative errors, and non-perseverative errors. *T*-Scores ≤ 44 are considered at the low limits of the norm.

Neuropsychological test administration and analysis of results were independently carried out in a single-blind manner by two pediatric neuropsychiatrists with 10 years of specific experience. Care was taken to ensure that each neuropsychiatrist tested an equal number of patients in both groups.

### 2.3. MRI Data Acquisition

All brain MRI studies were performed on the same 3T Scanner (Trio, Siemens Medical Systems, Erlangen, Germany) and included a 3D T1-weighted magnetization prepared rapid acquisition gradient echo sequence (MPRAGE; TE = 3.4 ms; TR = 1900 ms; TI = 900 ms; flip angle = 9°; FOV = 250; slice thickness = 1 mm; voxel dimension = 0.98 × 0.98 × 1.00 mm^3^; 160 axial slices) for structural analysis and a T2*-weighted echo-planar sequence (TR = 2500 ms; TE = 40 ms; FOV = 192 mm; 64 × 64 acquisition matrix; 30 axial slices; slice thickness = 4 mm; gap = 1 mm) for RS-fMRI analysis.

### 2.4. MRI Data Analysis

To assess possible regional differences in GM volume between the two groups, a VBM analysis was carried out. For this purpose, structural data were processed using the Statistical Parametric Mapping software package (SPM12, Wellcome Trust Center for Neuroimaging, University College London) [27]. Preprocessing steps included spatial registration of T1-weighted volumes to a reference brain template in Montreal Neurological Institute (MNI) space [28] with a fast diffeomorphic registration algorithm (diffeomorphic anatomical registration using exponentiated lie algebra, DARTEL) [29], tissue segmentation in GM, WM, and cerebrospinal fluid (CSF), and bias correction of intensity nonuniformities. Normalized modulated GM images were spatially smoothed using a 4 mm full width at half maximum (FWHM) isotropic Gaussian kernel [30]. Lastly, total intracranial volume (TIV) was estimated using non-normalized segmented volumes as the number of voxels where the sum of the three segmented tissue probabilities (GM, WM, and CSF) exceeded 50%.

RS-fMRI data were processed using a toolbox for FC analysis (CONN, v.16.a, McGovern Institute for Brain Research, Massachusetts Institute of Technology) [31], which contains libraries for fMRI analysis based on SPM12. Preprocessing steps included removal of the first five timepoints (to reduce the initial instability of MRI signal), motion correction, slice timing correction, temporal despiking, and spatial smoothing (using a 5 mm Gaussian kernel). From the motion correction procedure, the mean displacement of the brain voxels was computed as the root mean square (RMS) [32] of the translations along the three axes. Studies with a mean RMS of 0.5 mm or higher, more than 1.5 mm displacement along, or 1.5° rotation around any axis at any timepoint were discarded. Moreover, a “scrubbing” procedure was applied [33] to remove the timepoints, along with the preceding and the two following ones, that showed a framewise differential of signal intensity > 9 *z*-values, to reduce the effect of patient movements. Resulting data were then normalized to the standard Montreal Neurological Institute (MNI) echo-planar image template and resampled to a voxel size of 2 × 2 × 2 mm^3^. For each subject, the BOLD signal time course was calculated separately for the medial prefrontal cortex (MPFC) and posterior cingulate/precuneus (PCC), the main anterior and posterior hubs of the DMN. The MPFC and PCC ROIs available in CONN were used, which were obtained by independent component analysis of 497 normal subjects from the human connectome project dataset [34], masked for each patient by the corresponding GM map obtained by segmentation of the T1-weighted volume for the VBM analysis. 

For each ROI, the corresponding correlation map of the BOLD signal across the brain was generated, including in a general linear model (GLM) the time course of WM and CSF signals and the six parameters (translation and rotation along the *X*-, *Y*-, and *Z*-axes) of the spatial transformation, as derived from the co-registration step to remove the effect of residual motions on the echo-planar image signal. 

### 2.5. Statistical Analysis

The results of all neuropsychological tests from the total patient group were compared with published reference values [23,24,25,26], to assess the percentage of patients achieving a score that were at or below the low limits of the norm. Scores between groups were compared using the paired *t*-test for comparison between mean scores in paired samples and the Fisher’s exact test for comparison between proportions. A *p*-value < 0.05 was considered statistically significant.

For the VBM analysis, normalized and modulated GM maps were statistically analyzed using the GLM. To this aim, age and sex were included in a regression analysis (AnCova) as confounding variables to remove their effect on brain volume. TIV was also entered in the model to normalize to the head size. The analysis was limited to a mask-defined thresholding at 0.2 for the mean of the normalized GM maps, to further reduce the influence of non-encephalic tissue.

FC maps were statistically analyzed, separately for each of the two seeds, to test for possible differences between the two groups. When significant differences between the two groups emerged, the correlation of the corresponding seed(s) with the scores of WAIS/WISC-IV, WCST, and d2-R that showed values at or below the lower limit of the norm in ≥50% of the patients, or that showed significant differences between the two patient groups were assessed voxel-wise by AnCova in SPM12, including the group as a nuisance covariate. The inclusion of the group as nuisance in the model allowed assessing if the FC in that region correlated with the clinical score independent of group membership (correlation over the whole sample without group as nuisance would be noninformative, resulting in an obvious correlation as both the cluster FC and the clinical scores are selected as significantly different between the two groups). When comparing the two groups, both contrasts (Group A > Group B and Group B > Group A) were tested, whereas for correlations between FC and neurocognitive scores, both direct and inverse correlations were evaluated. Results of all voxel-wise analyses were considered significant for *p* < 0.05, corrected for “family-wise error” (FWE) at the cluster level, using a cluster-forming threshold of *p* < 0.001.

## 3. Results

### 3.1. Neuropsychological Scores 

#### 3.1.1. Wechsler Scale

The scores obtained are summarized in Table 2. Digit span scores were <1 SD in 76% of all patients, and Group A performed significantly worse than Group B in this subitem (*p* = 0.023) and for the digit symbol test (*p* = 0.013). All other scores were within the normal limits in >50% of the patients, without statistically significant differences between the two groups.

#### 3.1.2. D2-R Test of Attention

The results are depicted in Table 3; 17/26 (65.4%) and 14/26 (53.8%) patients scored <94 for processing speed and processing accuracy, respectively, without statistically significant differences between the two groups.

#### 3.1.3. Wisconsin Card Sorting Test

Table 4 summarizes the results. No score was <44 in more than 50% of patients. There were no significant differences between the two groups.

### 3.2. VBM

The VBM analysis of GM and WM did not show clusters of statistically significant differences in regional tissue volumes between the two groups.

### 3.3. RS-fMRI

#### 3.3.1. Between-Group FC Differences

The FC of the two seeds was overall consistent with the known pattern of positive and negative correlations of the anterior and posterior parts of the DMN [35], as shown in Supplementary Figures S1–S4.

**Figure 1:**
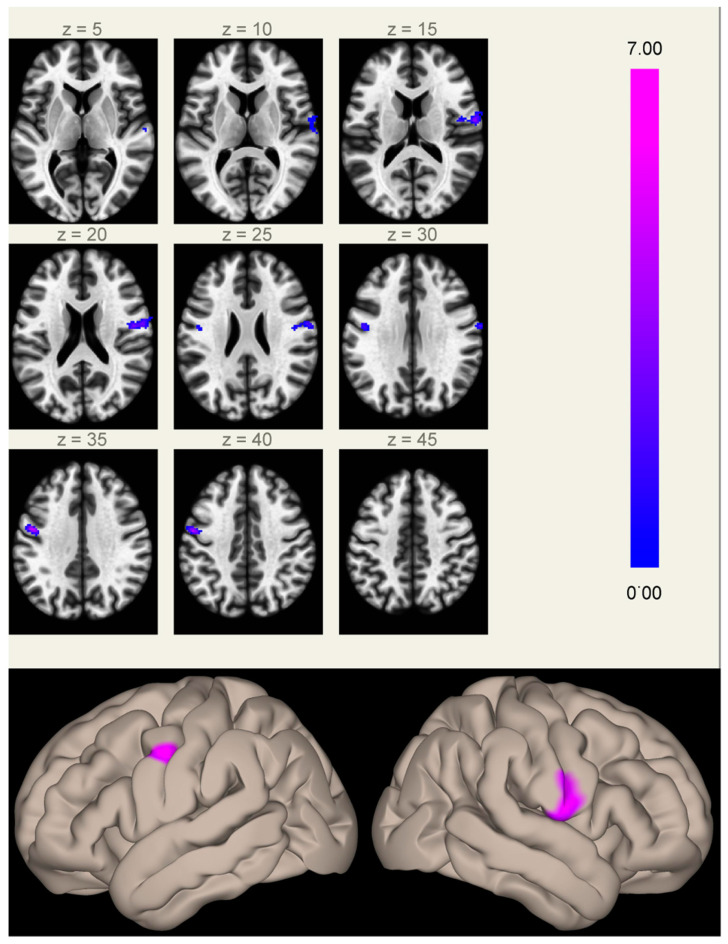
Clusters of increased correlation with the MPFC (anterior hub of the default mode network) in Group A compared to Group B. Significant clusters are located in the inferior part of the right (2.8 cm^3^, MNI coordinates of cluster maximum [+60 −06 +16]) and left (1.4 cm^3^, [−48 −04 +34]) precentral gyri. The color scale represents *T*-values from the GLM.

When comparing FC maps between the two groups, we found significantly increased FC between the MPFC (anterior hub of the DMN) and the inferior part of the right (2.8 cm^3^, *p* < 0.001 FWE-corrected at cluster level) and left (1.4 cm^3^, *p* < 0.05 FWE-corrected at cluster level) precentral gyri in Group A compared to Group B (Figure 1 and Figure S1). These clusters lie in regions normally devoid of significant connectivity with the DMN hubs, located between the two main regions anti-correlated to the DMN (the frontal eye field and the intraparietal sulcus), part of the task-positive network/dorsal attention network [19,36].

The FC of PCC (representing the posterior part of DMN) did not show any statistically significant difference between the two groups.

#### 3.3.2. Correlations between FC and Neurocognitive Tests

As only MPFC showed significant connectivity differences between the two groups, only this seed was probed for FC correlations with the four scores that showed abnormal results or significant differences between the two groups (digit span and digit symbol from WISC-IV/WAIS-R, and speed of processing and processing accuracy from d2-R). 

**Figure 2:**
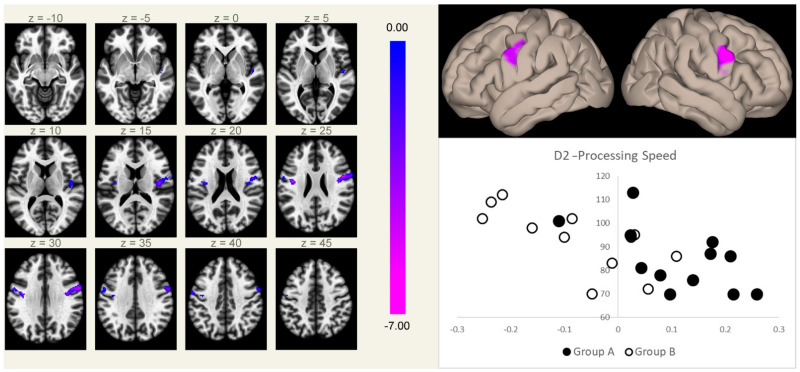
Connectivity between the MPFC and the right (5.9 cm^3^, MNI coordinates [+50 +02 +26]) and left (2.3 cm^3^, MNI coordinates [−40 −08 +24]) rolandic opercular cortices (a region normally devoid of physiological correlation with the MPFC) correlated inversely with the speed of processing scores at the d2-R test, independent of the group membership. Clusters of significant inverse correlation are shown overlaid on the axial images from a standard T1-weighted volume in the MNI space (left, in neurological convention, MNI Z coordinates are reported for each slice), and projected onto its surface (right, upper panel). The color scale represents *T*-values from the GLM. In the lower right box, the processing speed scores (*Y*-axis) are plotted against the mean Fischer-normalized correlation coefficients of the significant clusters (*X*-axis) for both groups (Group A, filled marks; Group B, empty marks).

The processing speed, as measured at the d2-R test of attention, showed a significant inverse correlation, independent of the therapy group, with the FC of the MPFC seed in two symmetric clusters centered on the precentral gyri, substantially overlapping the same regions that showed significant differences in FC with the MPFC between the two groups (Figure 2).

## 4. Discussion

In the last decades, the number of ALL LTSs has grown significantly due to the increased efficacy of the therapy [37]. Unfortunately, this major achievement is linked to a higher risk of developing secondary diseases and therapy side-effects [38]. Previous studies indeed report that ALL LTSs treated with ChT have lower performance in multiple domains of intelligence, academic achievement, processing speed, verbal memory, executive functioning and fine motor skills, compared to healthy controls [13,39]. The correlation between cognitive impairment and ALL pharmacological therapy is further supported by the correlation that has been proven between plasma concentrations of antimitotic drugs and cognitive impairment in ALL LTSs [40].

In line with the increased severity of cognitive impairment found in survivors who have undergone higher intensity therapy schemes, including RT [10], we found significantly lower scores at digit span and digit symbol tests in patients treated with ChT and RT versus those cured with ChT only.

In addition, we found differences in the functional connectivity of the DMN between the two patient groups, along with the absence of significant differences in terms of GM or WM volumes.

Regarding VBM results, differences in regional GM density between the two groups could be expected, as structural alterations have been previously reported in ALL LTSs. Brain tissue diffuse damage is typical of ALL LTSs and involves both GM and WM. The cost consistent findings are hippocampal atrophy [41,42,43,44] and a reduction in frontal and temporal WM volumes [10,45]. Further to this, a study on a large sample of ALL LTSs showed a significant effect of treatment intensity, including the inclusion of RT in the treatment protocol, on the WM loss [10].

The lack of volumetric differences at VBM analysis between our two ALL LTS subgroups suggests that the cerebral damage may similarly involve brain tissues in the two groups, despite the different therapeutic regimens used. From this standpoint, it should also be considered that brain tissue volume differences between the two subgroups, if present, are expected to be subtler than those found when comparing patients with healthy controls, as structural brain alterations have also been detected in patients treated by ChT only [39,46].

Our VBM results must be considered cautiously, given the relatively limited number of patients. Indeed, contrary to our results, adding RT to the therapeutic protocol has been previously correlated with more severe hippocampal damage, demonstrated by a dedicated ROI analysis [43]. However, the older age of our patients, compared to that of the patients described by Zając-Spychała, coupled to the differences in the analysis techniques (brain-wide voxel-based analysis versus ROI-based GM analysis with a priori hypothesis), may explain this discrepancy.

On the other hand, consistent to our findings, no effect due to the treatment has been linked to cortical surface area/thickness differences [47], further suggesting that structural differences between patients treated with ChT/RT and ChT-only, if present, are minimal.

Regarding FC results, we found increased connectivity between the MPFC (the anterior hub of the DMN) and the inferior part of both precentral gyri in patients treated with ChT and RT, compared to those treated with ChT only. Notably, the FC in the same regions correlated with processing speed scores, independent of the treatment group, while the FC of PCC, the main posterior hub of the DMN, did not show statistically significant differences between the two groups.

FC changes have been previously demonstrated in adult survivors of ALL treated with ChT [21,22,46]. To the best of our knowledge, however, no studies have been performed to assess the differences in FC and the relationship between FC and the cognitive performances in patients treated with ChT only or with ChT and RT.

In our study, FC differences emerged between the two groups of ALL LTS patients, albeit with the absence of significant differences in terms of brain tissue volumes. In particular, an increased correlation between MPFC and the rolandic opercula was present in patients treated with both ChT and RT, as compared to those treated with ChT alone.

Of note, these FC differences may play a role in determining differences at the digit span and digit symbol test scores between the two groups. Interestingly, the relevance of the rolandic operculum to working memory (probed by the digit span test) was witnessed by lesion studies. Using the voxel-based lesion-symptom mapping approach, Glascher et al. described a statistically significant lesion-deficit relationship between the rolandic operculum and working memory [48]. These findings represent a supporting background to the present results. 

Patients with lower scores in the d2-R test of attention showed two clusters of significantly higher FC, reaching positive slope values in Group A, with the MPFC, independently from the differences in both FC and score due to the treatment group. These two clusters substantially overlap those that emerged from the between-group contrast, and fall in a region normally devoid of FC with the DMN at the center of the two main regions anti-correlated to the DMN [19,36]. These data suggest that changes in connectivity between these two regions occur in both groups to a different extent and either may be responsible for the decrease of the neurocognitive performance or more probably may represent inadequate compensatory mechanisms.

Since this correlation was independent of the type of treatment (RT + ChT or ChT alone), we hypothesize that RT can to some extent act as a strengthener of the same noxious mechanisms induced by ChT. Indeed, alteration of FC among networks has been reported in ALL LTSs treated with ChT alone, and it could be related to a connectome disruption as it is associated with delayed neurodevelopment [22]. In these patients, lower modularity of both the structural and the functional connectomes was present in subjects with executive dysfunction, indicating lesser separation among networks, consistent with hyperconnectivity [22]. Thus, we can speculate that these FC changes may be due to a higher burden of damage of the brain determined by the combination of ChT and RT, compared to ChT alone. In particular, the persistent effects of RT on the proliferation of oligodendrocytes and/or progenitor cells, affecting myelination and restorative capacity of the CNS [49], may play a role in this respect.

Alternatively, these FC changes may represent compensatory mechanisms actuated by the brain in an attempt to overcome the damage, as described for other pathological conditions such as visual and hearing loss [48,50].

In addition to the potential causative role of the therapy, it must be considered that the disease in itself may also play a role in FC alterations. Hu et al. described changes in local FC involving the DMN and the precentral gyrus among children with ALL at disease onset, before any treatment had been carried out, suggesting that this kind of dysfunction could also derive directly from the pathology [51]. Interestingly, this finding was later further confirmed and integrated by the same group, showing a beneficial effect of treatment on local FC, which was at least partly normalized by effective therapy [52]. The increase in internetwork connectivity, with the appearance of spurious connectivity between a DMN hub and regions traditionally not included in the DMN (including the precentral gyrus), may be related to the disruption of local homogeneity in these regions [49,51,52]. From this standpoint, it cannot be excluded that the alterations detected in our patients may represent a therapy-related enhancement of FC changes that are already caused by the disease. Longitudinal studies are needed to test this hypothesis.

Several limitations must be taken into account when considering the current results.

The lack of healthy controls for comparison did not allow precise framing in a region belonging to a specific network the significant clusters, nor assessing if the correlation between FC and d2-R scores that we found in these patients is the strengthening of a correlation present already under normal conditions.

In addition, the limited sample, coupled to the predicted diversity of participants and the noise inherent to both the RS-fMRI data and the neuropsychological scores, renders the achievement of a definitive conclusion difficult. Indeed, it should be considered that, although definitely larger sample sizes are mainly required to avoid false negatives, limited sample size is also associated to false positives, even when using as in our case a stringent (*p* = 0.001) cluster-forming threshold [53], while avoiding the false discovery rate approach in favor of FWE correction for multiple comparisons, which is less prone to false positives [54], coupled to a rigorous control for motion. However, we argue that the substantial symmetry of the results and the simultaneous presence in the two symmetrical clusters of a difference in FC between the two groups and of a correlation between the FC and the neuropsychological score, independent of the difference between groups, provide evidence against the hypothesis of a casual origin, related to the small sample size of our findings.

Lastly, we did not control for the intensity of the chemotherapy performed in each patient. This is of particular concern, considering the different risk profile (higher in Group A) eligible for the two treatment strategies. However, we consider this difference unlikely to have caused a diverse late toxicity profile, in view of the pattern of administration of methotrexate injections (the unique chemotherapeutic agent included in these protocols which is able to cross the BEE) and the total number of intrathecal injections in the two groups. In particular, the higher risk profile resulted in a reduced number of intrathecal administrations of methotrexate in Group A (Supplementary Table S2), as indicated to limit RT-related neurotoxicity by both AIEOP-ALL 95 and AIEOP-BFM-ALL 2000 therapeutic protocols. The reduced number of intrathecal methotrexate administrations in Group A, however, actually reinforces the hypothesis that differences seen in these patients are induced by the adjunct of radiotherapy. On the other hand, while only a minor difference between the two groups in terms of intravenous methotrexate was present (total dose 10.8 ± 2.8 gr/m^2^ in Group A vs. 9.1 ± 3.3 gr/m^2^ in Group B), a less tight administration schedule in Group B may have also played a role, albeit unlikely to overcome possible differences related to the different intrathecal doses.

Accordingly, additional studies with larger sample size, including a control group, are warranted to confirm and expand the present results.

## 5. Conclusions

We demonstrated, in ALL LTSs treated by combined ChT and RT, compared to those who underwent ChT only, significantly lower neurocognitive scores probing working memory and speed of processing, coupled to an increased FC of the anterior part of the DMN with the precentral cortex at the level of the rolandic opercula. This FC increase, which is not associated with significant regional structural alterations, shows a significant inverse correlation with the scores of neurocognitive tests that probe processing speed. Further longitudinal studies are needed to clarify the significance of these changes, which may represent either a direct expression of the mechanisms underlying the cognitive deficits or ineffective compensatory phenomena.

## Figures and Tables

**Table 1:** Demographics of the enrolled patients.

	TOT	Group A	Group B	*p*-Value
Number	26	13	13	
Age (years, mean ± SD)	17.66 ± 3.72	17.87 ± 4 04	17.44 ± 3.51	0.47 ^#^
Schooling (years, mean ± SD)	11.66 ± 3.72	11.87 ± 4.04	11.44 ± 3.51	0.49 ^#^
Sex	18 M/8 F	9 M/4 F	9 M/4 F	1.00 ^§^
Hand dominance (right/left)	25/1	13/0	12/1	1.00 ^§^
Glasses (yes/no)	19/7	10/3	9/4	1.00 ^§^
Age at diagnosis (years, mean ± SD)	5.02 ± 4.12	5.29 ± 4.53	4.75 ± 3.83	0.15 ^#^
Off therapy (years, mean ± SD)	10.67 ± 4.18	10.62 ± 4.33	10.73 ± 4.20	0.67 ^#^
Therapy protocol (AIEOP 95/AIEOP 00)	10/16	5/8	5/8	1.00 ^§^

**^#^** Significance at paired *t*-test; **^§^** significance at Fischer’s exact test.

**Table 2:** Results of the WAIS-R/WISC-IV scales in the patients of the study.

		TOT	Group A	Group B	*p*-Value
Total intelligence quotient	mean	95.23	90.62	99.85	0.097
	SD	15.36	13.39	16.31	
Digit span	mean	6.31	5.08	7.54	0.023
	SD	2.59	3.14	2.47	
Digit symbol	mean	7.38	5.92	8.85	0.013
	SD	2.94	2.22	2.91	
Block design	mean	10.46	9.46	11.46	0.164
	SD	3.28	3.13	3.23	
Vocabulary	mean	9.85	9.69	9.85	0.874
	SD	2.77	2.53	3.02	
Comprehension	mean	11.19	11.15	11.15	1.000
	SD	2.06	2.41	1.68	
Similarities	mean	10.54	10.23	10.69	0.475
	SD	2.02	1.69	2.39	

Results of the tests of the WAIS-R/WISC-IV scales in the patients of the study. According to the normative reference values, total intelligence quotient normal values are included between 90 and 109, while the normal ranges for subtest scores are 10 ± 2. Digit span scores were <1 SD in 76% of all patients. All the other scores were within the norm in >50% of the patients. For each test, the significance of the differences between the two study groups (Group A = chemotherapy and radiotherapy, Group B = chemotherapy only) at paired *t*-test is reported.

**Table 3:** Results of D2 test of attention in the patients of the study.

		TOT	Group A	Group B	*p*-Value
Speed of processing	mean	88.30	85.30	91.31	0.35
	SD	13.70	13.00	14.20	
Processing accuracy	mean	93.10	90.85	95.31	0.39
	SD	12.80	13.35	12.40	
Error rate (%)	Mean	99.80	98.62	101.08	0.52
	SD	10.60	13.19	7.47	

Results of the D2R scale subitems in the patients of the study. Results are reported as a percentage of normative data. For each subitem, the significance of the differences between the two study groups (Group A = chemotherapy and radiotherapy, Group B = chemotherapy only) at paired *t*-test is reported.

**Table 4:** Results of the WCST scale in the patients of the study.

		Tot	Group A	Group B	*p*-Value
Total number of errors	mean	51.00	49.23	52.77	0.58
	SD	15.20	13.10	17.40	
Perseverative errors	mean	53.70	51.39	56.00	0.84
	SD	12.60	13.37	11.10	
Non-perseverative errors	mean	52.00	53.23	50.69	0.66
	SD	18.00	18.59	18.02	

Results of the Wisconsin Card Sorting Test subitems in the patients of the study. For each subitem, results are expressed as *T*-scores, and the significance of the differences between the two study groups (Group A = chemotherapy and radiotherapy, Group B = chemotherapy only) at paired *t*-test is reported.

## Data Availability

The data presented in this study are available upon reasonable request for justified research purposes from the corresponding author. The data are not publicly available for confidentiality reasons.

## Supplementary Materials

The following supporting information can be downloaded at https://www.mdpi.com/article/10.3390/biology11040499/s1, Figure S1. FC maps of the MPFC seed in Group A; Figure S2. FC maps of the MPFC seed in Group B; Figure S3. FC maps of the PCC seed in Group A; Figure S4. FC maps of the PCC seed in Group B; Supplementary Table S1. Characteristics of the disease at the onset; Table S2. Details of treatments [55,56].
