# Supplementary material for: Default-Mode Network Connectivity Changes Correlate with Attention Deficits in ALL Long-Term Survivors Treated with Radio- and/or Chemotherapy
Source: Biology (Basel). 2022 Mar 24;11(4):499. doi: 10.3390/biology11040499 (PMC9024843; doi:10.3390/biology11040499)
Supplement: Supplementary file 1 [file biology-11-00499-s001.zip › biology-1593291-supplementary.pdf]

Supplementary Material

Default-Mode Network connectivity changes correlate with attention deficits in ALL long-term survivors treated with radio- and/or chemotherapy.

Federica Mazio [1,§], Giuseppina Aloj [2,§], Teresa Perillo [3], Grazia Maria Giovanna Pastorino [4], Carmela Russo [1], Maria Pia Riccio [5,] Eugenio Maria Covelli [1], Rosanna Parasole [2], Enrico Tedeschi [3], Lorenzo Ugga [3], Alessandra D'Amico [6,#], Mario Quarantelli [7,#,*]

[§] **These authors share first authorship**
[#] **These authors share senior authorship**

[1] Pediatric Neuroradiology, Department of Neuroscience, Santobono-Pausilipon Children's Hospital, Naples, Italy; federicamazio1@gmail.com (FM), russocarmela84@gmail.com (CR), e.covelli@santobonopausilipon.it (EMC)
[2] Department of Pediatric Hemato-Oncology, A.O.R.N. Santobono-Pausilipon, Naples, Italy; giuseppinaaloj@gmail.com (GA), rparasol64@gmail.com (RP)
[3] Department of Advanced Biomedical Sciences, University of Naples Federico II; tperillo3@gmail.com (TP), enrico.tedeschi@unina.it (ET), lorenzo.ugga@gmail.com (LU)
[4] Child and Adolescent Neuropsychiatry Unit, Department of Medicine, Surgery and Dentistry, University of Salerno, Salerno, Italy; graziapastorino@gmail.com (GMGP)
[5] Department of Medical and Translational Sciences, Child Neuropsychiatry, Federico II University, Via Pansini 5, Naples, Italy; piariccio@gmail.com (MPR)
[6] Department of Radiology, Tortorella Private Hospital, Salerno, Italy; damicoalex@tiscali.it (Ad'A)
[7] Institute of Biostructure and Bioimaging, National Research Council, Naples, Italy; quarante@unina.it (MQ)

*Correspondence: Mario Quarantelli, MD; quarante@unina.it (MQ)

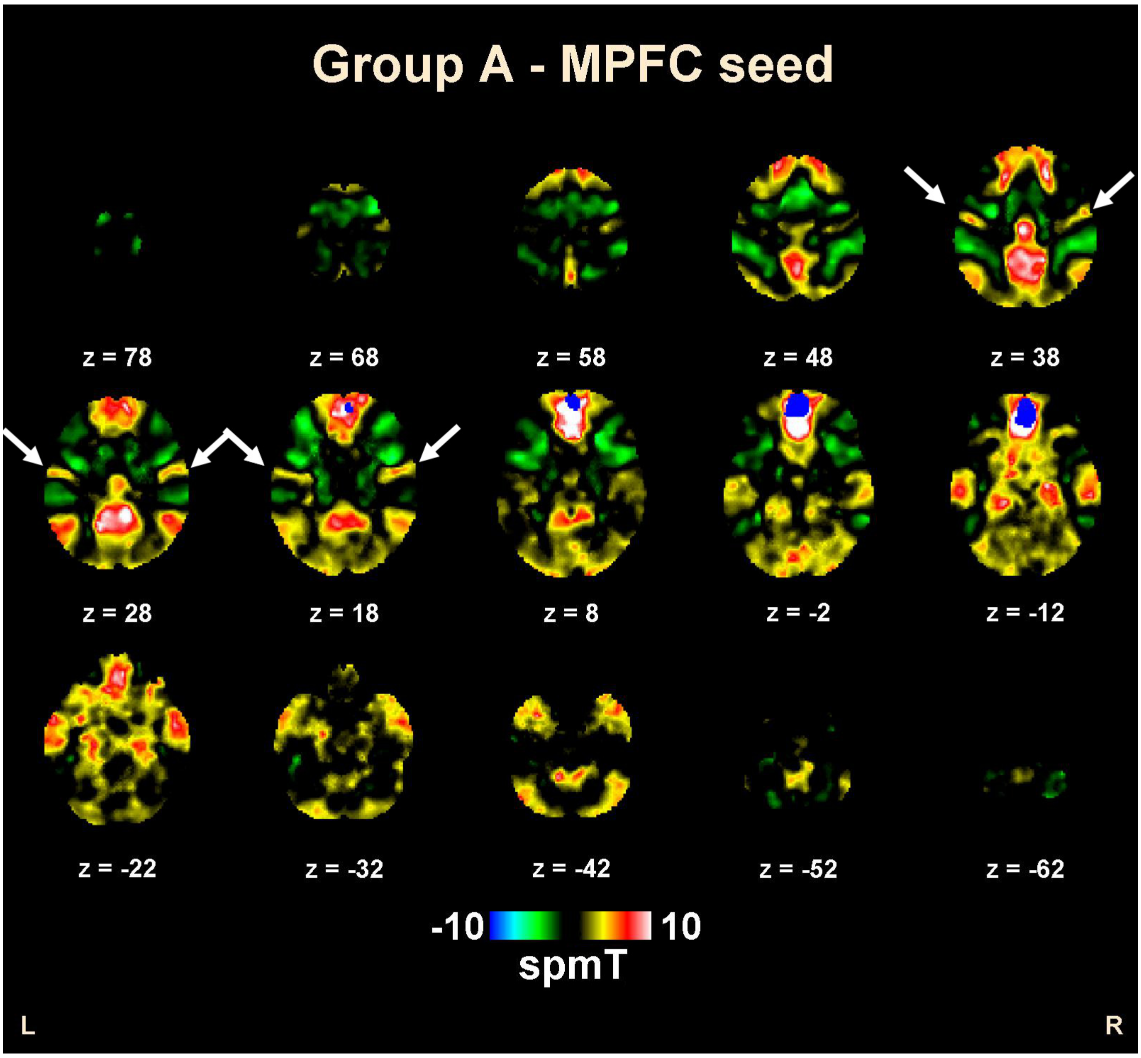

Figure S1. Axial planes (MNI z coordinates in mm are reported) from the map of the mean functional connectivity of the MPFC seed (in blue) in patients treated with both chemo- and radiotherapy.

The overall pattern is consistent with the known configuration of the DMN, with characteristic main local maxima in the MPFC, PCC, inferior parietal lobes bilaterally, medial temporal lobe and cerebellar tonsils.

Units are t values derived from the GLM. Two clusters of positive correlation in the rolandic opercular cortices (arrows) are present in a region where no correlation or mild anticorrelation is present in Group B patients (see Figure S2), corresponding to the region where significant clusters highlighted in Figures 1 and 2 are located.

Color scale represents T values from the GLM.

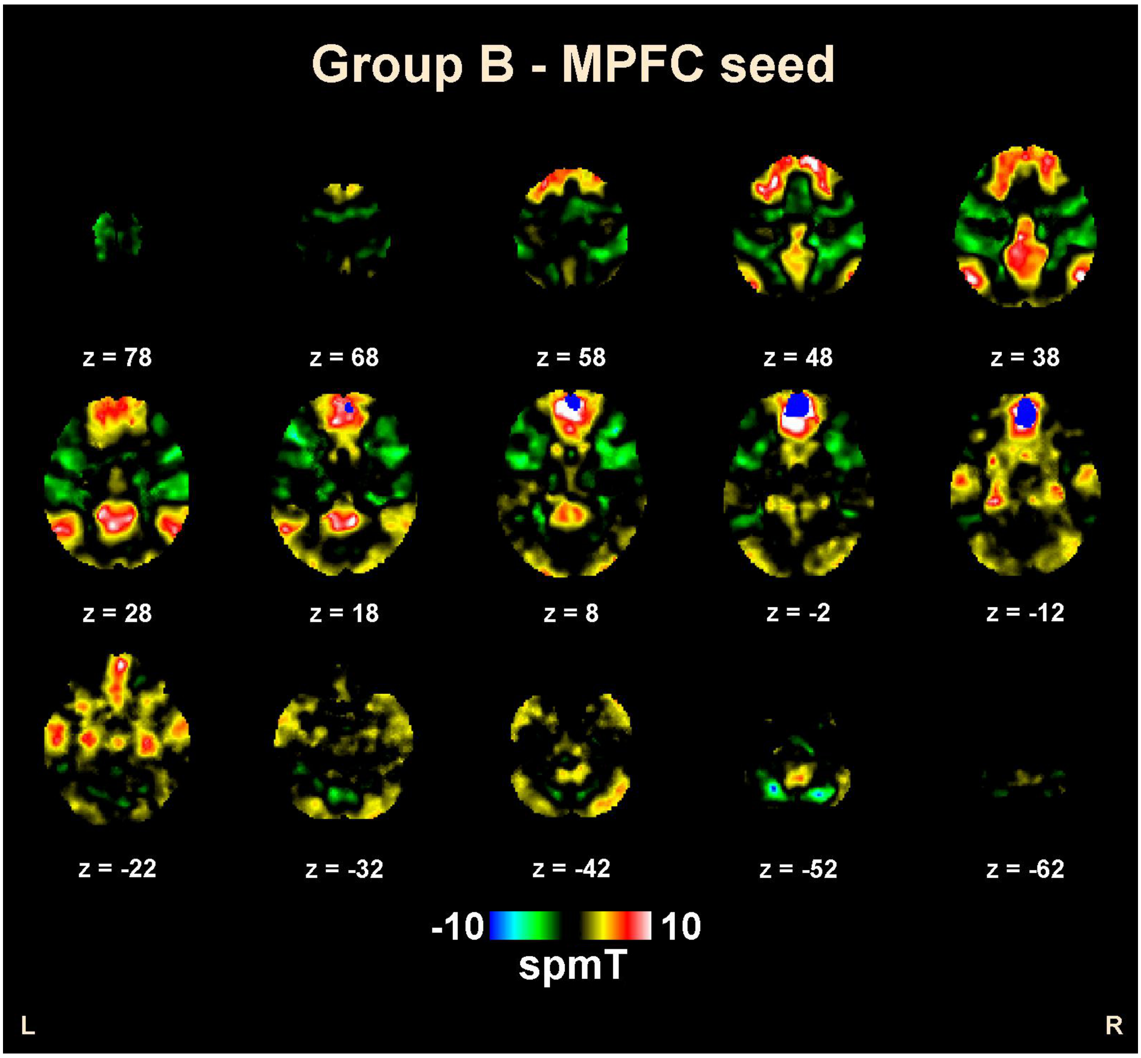

Figure S2. Axial planes (MNI z coordinates in mm are reported) from the map of the mean functional connectivity of the MPFC seed (in blue) in patients treated only with chemotherapy.

The overall pattern is consistent with the known configuration of the DMN, with characteristic main local maxima in the MPFC, PCC, inferior parietal lobes bilaterally, medial temporal lobe and cerebellar tonsils.

Color scale represents T values from the GLM.

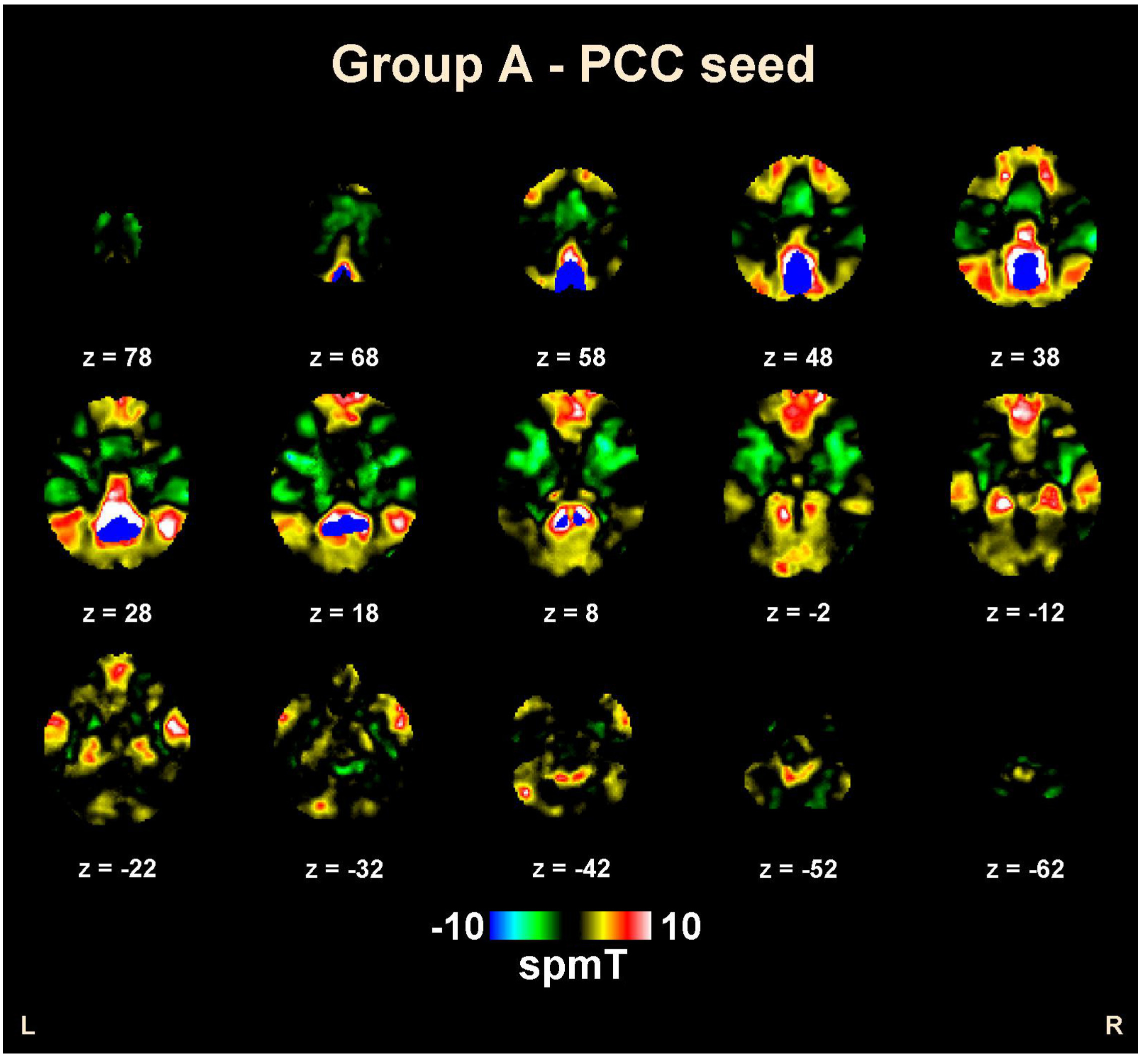

Figure S3. Axial planes (MNI z coordinates in mm are reported) from the map of the mean functional connectivity of the PCC seed (in blue) in patients treated with both chemo- and radiotherapy.

The overall pattern is consistent with the known configuration of the DMN, with characteristic main local maxima in the MPFC, PCC, inferior parietal lobes bilaterally, medial temporal lobe and cerebellar tonsils.

Color scale represents T values from the GLM.

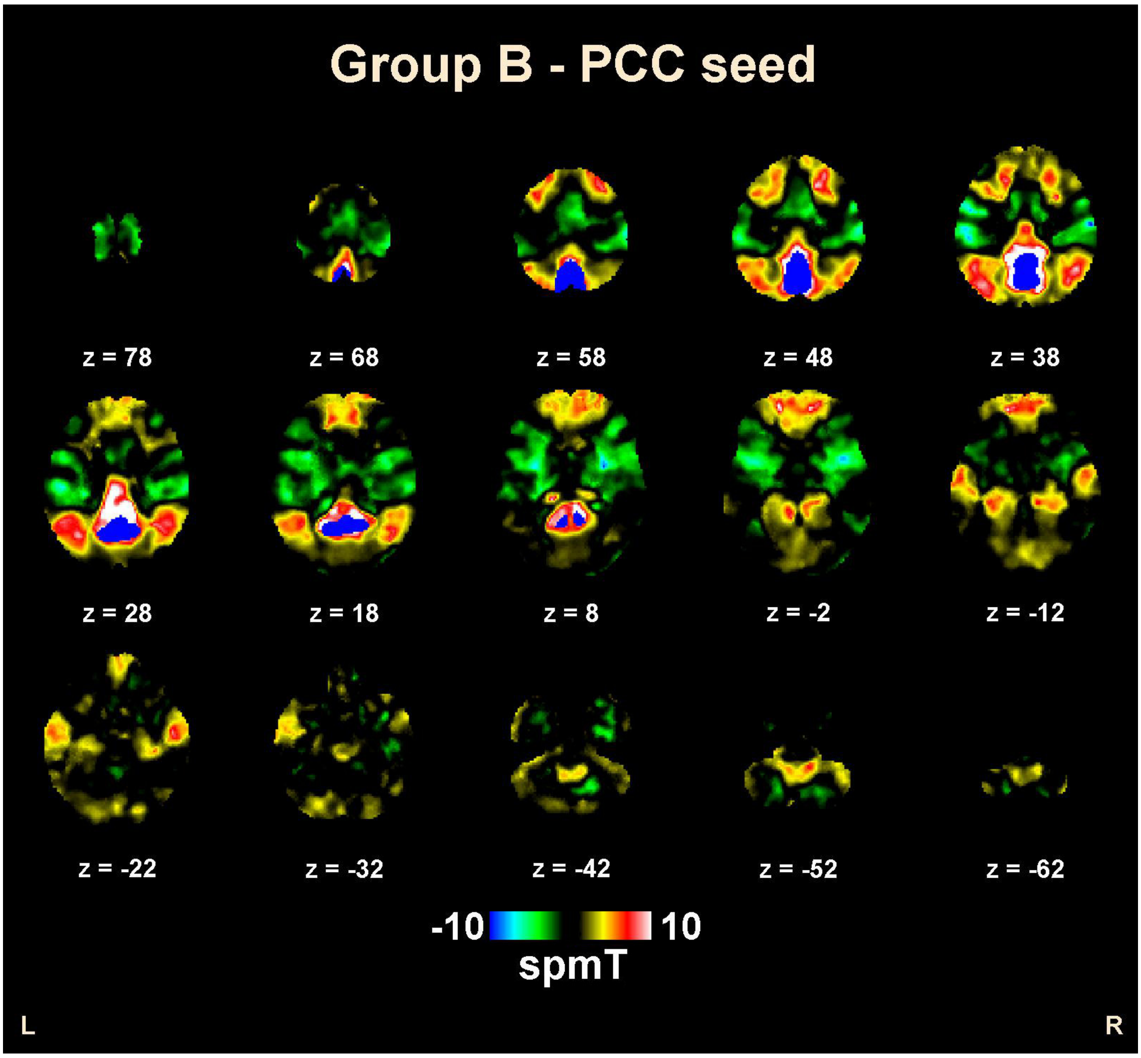

Figure S4. Axial planes (MNI z coordinates in mm are reported) from the map of the mean functional connectivity of the PCC seed (in blue) in patients treated with chemotherapy only. The overall pattern is consistent with the known configuration of the DMN, with characteristic main local maxima in the MPFC, PCC, inferior parietal lobes bilaterally, medial temporal lobe and cerebellar tonsils.

Color scale represents T values from the GLM.

Table S1. Characteristics of the disease at the onset.

	Group A	Group B
**ALL Subtype T**	4 pts	1 pts
**ALL Subtype B**	9 pts	12 pts
**Karyotype**<br>• t(12;21)<br>• t(1;19)<br>• Complex karyotype<br>• No alterations	 2 pts<br>0 pts<br>2 pts<br>9 pts	 2 pts<br>2 pts<br>2 pts<br>9 pts

All the subtype B ALL patients were of the common
No patient had pro-B ALL Subtype.

Table S2. Details of treatments

		Group A	Group B
**AIEOP-ALL 95** [1]		5 pts	5 pts
**AIEOP-BFM-ALL 2000** [2]		8 pts	8 pts
**High Risk Stratification**		12 pts	1 pt
**Medium / Standard Risk Stratification**		1 pt	12 pts
**MTX HD 5 gr/m2**	**4 doses**	1 pt	1 pt
	**2 doses**	12 pt	1 pt
**MTX HD 2 gr/m2**	**4 doses**	No pts	11 pts
**Intrathecal MTX + MP**		8 pts (14 - 17 injections)	8 pts (18-22 injections)
**Intrathecal MTX + Cytarabine + MP**		5 pts (10 injections)	5 pts (16-18 injections)
**RT 24 Gy**		1 pt	n/a
**RT 18 Gy**		12 pts	

MTX: Methotrexate
MP: Methylprednisolone
RT: Radiotherapy

References

1. Aricò, M., Valsecchi, M.G., Rizzari, C., Barisone, E., Biondi, A., Casale, F., Locatelli, F., Lo Nigro, L., Luciani, M., Messina, C., Micalizzi, C., Parasole, R., Pession, A., Santoro, N., Testi, A.M., Silvestri, D., Basso, G., Masera, G., Conter, V., 2008. Long-term results of the AIEOP-ALL-95 Trial for Childhood Acute Lymphoblastic Leukemia: insight on the prognostic value of DNA index in the framework of Berlin-Frankfurt-Muenster based chemotherapy. J. Clin. Oncol. 26, 283–289. https://doi.org/10.1200/JCO.2007.12.3927
2. Möricke, A., Zimmermann, M., Valsecchi, M.G., Stanulla, M., Biondi, A., Mann, G., Locatelli, F., Cazzaniga, G., Niggli, F., Aricò, M., Bartram, C.R., Attarbaschi, A., Silvestri, D., Beier, R., Basso, G., Ratei, R., Kulozik, A.E., Lo Nigro, L., Kremens, B., Greiner, J., Parasole, R., Harbott, J., Caruso, R., von Stackelberg, A., Barisone, E., Rössig, C., Conter, V., Schrappe, M., 2016. Dexamethasone vs prednisone in induction treatment of pediatric ALL: results of the randomized trial AIEOP-BFM ALL 2000. Blood 127, 2101–2112. https://doi.org/10.1182/BLOOD-2015-09-670729
